# The orphan response regulator EpsW is a substrate of the DifE kinase and it regulates exopolysaccharide in *Myxococcus xanthus*

**DOI:** 10.1038/srep17831

**Published:** 2015-12-07

**Authors:** Wesley P. Black, Lingling Wang, Manli Y. Davis, Zhaomin Yang

**Affiliations:** 1 Department of Biological Sciences, Virginia Polytechnic Institute and State University, Blacksburg, VA 24061, USA; 2 College of Life Sciences, South China Agricultural University, Guangzhou 510642, China

## Abstract

Here we attempted to identify the downstream target of the DifE histidine kinase in the regulation of exopolysaccharide (EPS) production in the Gram-negative bacterium *Myxococcus xanthus*. This bacterium is an important model system for the studies of Type IV pilus (T4P) because it is motile by social (S) motility which is powered by T4P retraction. EPS is critical for S motility because it is the preferred anchor for T4P retraction in this bacterium. Previous studies identified the Dif chemosensory pathway as crucial for the regulation of EPS production. However, the downstream target of the DifE kinase in this pathway was unknown. In this study, EpsW, an orphan and single-domain response regulator (RR), was identified as a potential DifE target first by bioinformatics. Subsequent experiments demonstrated that *epsW* is essential for EPS biosynthesis *in vivo* and that EpsW is directly phosphorylated by DifE *in vitro*. Targted mutagenesis of *epsW* suggests that EpsW is unlikely the terminal RR of the Dif pathway. We propose instead that EpsW is an intermediary in a multistep phosphorelay that regulates EPS in *M. xanthus*.

*Myxococcus xanthus* is a model bacterium for the studies of multicellular behavior in prokaryotes^1^,^2^. During vegetative growth, this bacterium moves as multicellular groups over solid surfaces. This is known as social swarming, which allows large groups of cells to move as a pack for the utilization of complex organics as nutrients in a cooperative manner^3^. Under starvation, hundreds of thousands of *M. xanthus* cells use their surface motility to aggregate and develop multicellular structures known as fruiting bodies^4^,^5^. Within these structures, cells of this Gram-negative bacterium eventually differentiate into metabolically dormant myxospores which are resistant to environmental stresses such as heat, desiccation and UV radiation. Both vegetative swarming and developmental aggregation are social behaviors that requires the collective action of many cells.

Integral to the social behavior of *M. xanthus* is its ability to move or glide over solid surfaces^1^,^5^,^6^. The motility of *M. xanthus* entails two genetically and mechanistically distinct systems known as adventurous (A) and social (S) motility^7^. The A-motility system allows cells to move even when they are well separated. The S system, which is the focus of this study, is only functional when cells are in close proximity. It is now known that S motility is powered by the retraction of the Type IV plius (T4P) as is bacterial twitching motility^8^. In the current model for T4P-dependent motility, the distal end of a T4P is first tethered to a solid surface. The ensuing retraction of the T4P pulls the cell forward to result in a net translocation over the surface. The tip of a *M. xanthus* T4P attaches preferentially to exopolysacchrides (EPS) produced by its kin. These EPS are either deposited on a gliding substratum by or associated with the cell surface of a neighboring *M. xanthus* cell. This mechanism explains the social nature of T4P-mediated motility in *M. xanthus* because other cells are typically required as a source of EPS to activate the retraction or the motor function of T4P.

EPS production is regulated in part by a pathway consisting of the Dif chemosensory proteins in *M. xanthus*^8^. DifA is homologous to the methyl-accepting chemotaxis protein (MCP), DifC to the bridging or coupling protein CheW and DifE to the CheA-type histidine protein kinase (HK)^9^. These three proteins, which form a membrane signaling complex^10^,^11^, are required for EPS production^12^. DifD and DifG, respective homologues of CheY and CheC, are not required for EPS production^13^. They are negative regulators of EPS instead because their absence results in an overproduction of EPS. DifD is a single-domain response regulator (RR) that can be phosphorylated by the DifE kinase^11^. Like CheC^14^,^15^, DifG is a phosphatase that accelerated the dephosphoryaltion of phosphorylated DifD (DifD~P)^11^. Interestingly, DifD does not function downstream of DifE in EPS regulation and it likely acts to divert phosphate from DifE as a phosphate sink^16^,^17^. This led to a model wherein *M. xanthus* DifE autophosphorylates on a strictly conserved histidine residue and the phosphate is then transferred to a conserved aspartate on a hypothesized RR directly downstream of DifE in EPS regulation. In other words, DifE is proposed to phosphorylate at least one additional RR besides its known target DifD.

There are known instances where a HK phosphorylates multiple RRs in two-component signal transduction pathways. In bacterial chemotaxis, a CheA kinase typically phosphorylates CheY as well as CheB, a methylesterase with a RR domain^18^,^19^. CheA also phosphorylates CheV in *Bacillus subtilis* and an additional CheY in S*inorhizobium meliloti*. In an extreme case in *Rhodobacter sphaeroides*, CheA3 interacts with five RRs in chemotaxis regulation. Such examples of one HK to multiple RRs are also found in canonical two-component systems^20^,^21^. The response regulators NarL and NarP are both substrates of the same HKs NarX and NarQ in the regulation of anaerobic respiration with nitrate and nitrate as electron acceptors^22^. In the cyanobacterium *Synechococcus elongatus*, NblS was found to phosphorylate both RpaB and SrrA in the regulation of photosynthesis and metabolism^20^,^23^. However, despite the many HKs and RRs in a cell, signal transduction pathways are well insulated from one another by specific interactions between HKs and RRs. That is, whether a HK has one or multiple cognate RRs, it only phosphorylates its target specifically and there is no evidence of nonspecific interactions between HKs and RRs^20^,^21^. The structural determinants of the high specificity between HKs and RRs have been studied extensively by co-variant analysis^24^,^25^,^26^,^27^,^28^. These analyses identified certain residues in RRs as critical determinants for their specific interaction with their cognate HKs. Mutations of these residues have been shown to alter and rewire the specificity between RRs with HKs^29^,^30^, validating bioinformatics as a useful approach to identify RRs, especially orphan ones, as target for specific HKs. In this study, DifD was used as a template to search for other RRs as potential targets of DifE phosphorylation by sequence analysis first. Subsequent studies both *in vivo* and *in vitro* led to the conclusion that EpsW, an orphant single-domain RR, is a target of DifE phosphorylation and part of the EPS regulatory pathway in *M. xanthus*.

## Results

### Bioinformatics analysis identified EpsW as a possible substrate of DifE kinase

**Figure 1:**
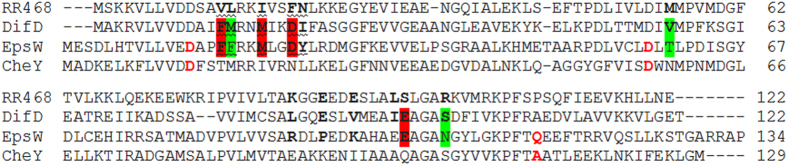
Alignment of RR4468, DifD, EpsW and CheY. The primary specificity-determinant residues in RR468, DifD and EpsW are in bold and underlined whereas the secondary ones are in bold only. The identical specificity-determinant residues in DifD and EpsW are highlighted in red and the conserved or semi-conserved ones in green. The three CheY residues in red can be mutated to generate constitutively active proteins and their counterparts in EpsW (also in red) were selected for specific mutagenesis in this study.

Previous co-variant studies^24^,^25^,^26^,^27^,^28^ indicated that certain residues in RRs are critical determinants of specificity with regard to their interactions with their cognate HKs. These residues in RRs can be divided into two sets with the first one consisting of five residues and the second one seven^27^. The first or the primary set contributes directly to the physical interactions between a RR-HK pair whereas the second does so indirectly. These residues as defined in RR468, a single-domain RR from *Thermatoga maritime*^31^, are shown in Fig. 1^27^. In this RR, the primary residues are V13, L14, I17, F20 and N21 and the secondary ones are M55, K85, E88, E91, L95, S96 and R100. DifD is a single domain RR that is phosphorylated by DifE^11^. Its specificity determinants were therefore used to identify additional DifE substrates. Alignment with RR468 indicated that the five primary residues in DifD (Fig. 1) are F13, M14, M17, D20 and I21 and the secondary ones are V56, L84, E87, V90, I94, E95 and S99.

Next, DifD was used to search the *M. xanthus* genome sequence^32^ producing alignment with over 100 RRs^33^. These RRs were manually enumerated to identify those with at least two identical residues with DifD in the primary group. This narrowed the number to eight ORFs. They are MXAN_2050, MXAN_4049, MXAN_4232, MXAN_4463, MXAN_5053, MXAN_6627 and MXAN_7396 and MXAN_7420. Among them, only MXAN7420 or EpsW^34^ matched with DifD (Fig. 1) three out of the five primary residues (F13, M17 and D20 in DifD; and F16, M20 and D23 in EpsW) with one additional conserved substitution (M14 in DifD and F17 in EpsW). For the seven secondary sites, DifD and EpsW share one identical residue (E85 in DifD and E101 in EpsW) and 2 somewhat conserved ones (V56 and S99 in DifD; T60 and N105 in EpsW) (Fig. 1). The observed conservation of specificity determinant residues here suggested one or more of these eight RRs, especially EpsW, could be substrates for DifE phosphorylation.

### *epsW* is required for EPS production

**Figure 2:**
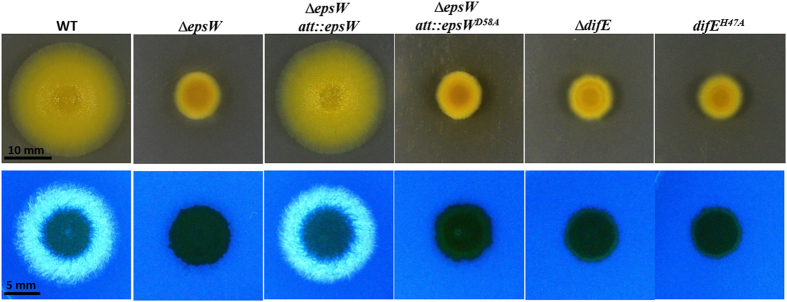
Examination of S motility and EPS production. Upper Panel. Examination of S motility on soft (0.4%) agar plates. Lower Panel. Analysis of EPS production on plates containing Calcofluor white. The genotypes of the strains are indicated on the top of the figure. The strains are DK1622 (wild-type, WT), YZ1830 (Δ*epsW*), YZ1831 (Δ*epsW att*::*epsW*), YZ1833 (Δ*epsW att*::*epsW*^*D58A*^), YZ603 (Δ*difE*) and YZ1369 (*difE*^*H47A*^). See Methods for details.

These eight RR genes were targeted for mutagenesis. *epsW* is a small gene encoding a single-domain RR while the other seven genes all encode larger proteins with additional domains^32^. As a consequence, an in-frame deletion was constructed for *epsW* whereas other RR genes were mutated by simple insertions. Except the *epsW* mutation, none of the others led to an EPS^-^ phenotype (data not shown) and this manuscript will only focus on *epsW* hereafter. As shown in Fig. 2, the Δ*epsW* mutant colony did not florescence on plates supplemented with a florescent dye as an EPS indicator (See Methods), showing that the Δ*epsW* mutant is defective in EPS production. In addition, this mutant was defective in swarming on soft (0.4%) agar, indicating S-motility deficiencies^35^. The wild-type *epsW* complemented Δ*epsW* in both S motility and EPS production assays (Fig. 2). These results demonstrate clearly that EpsW, a single-domain RR, plays an indispensable role in *M. xanthus* EPS regulation. It should be noted that *espW* was so designated because it is associated with known EPS biosynthetic genes at the *eps* locus^34^, but no *epsW* mutation had been constructed and examined previously. EpsW is considered an orphan RR because no HK gene is found in the immediate vicinity of *epsW*^34^.

**Figure 3:**
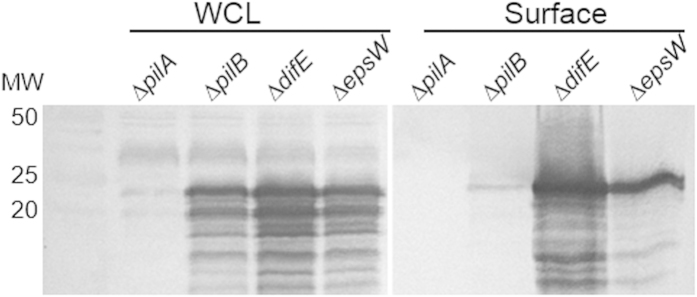
Examination of PilA expression and T4P assembly. To examine PilA expression, whole cell lysates (WCL) were prepared from 5 × 10^7^ cells. For the examination of T4P assembly, the fraction of surface pili (Surface) were prepared from 5 × 10^8^ cells as described in Methods. Samples were analyzed by immunoblot analysis using anti-PilA antibodies. The first lane contained molecular weight standard (MW) with the weight in KD indicated on the left. Strains analyzed were DK10407 (Δ*pilA*), DK10416 (Δ*pilB*), YZ603 (Δ*difE*) and YZ1830 (Δ*epsW*). See Methods for more details.

It is known that T4P functions upstream of the DifE pathway and T4P^-^ mutants are EPS^-^16,36,37. A possible explanation for the above observations (Fig. 2) was that the Δ*epsW* mutation eliminated or attenuated either T4P expression or assembly and this T4P defect in turn resulted in the observed EPS^-^ phenotype of the Δ*epsW* mutant. To examine this possibility, we tested whether the deletion of *epsW* affected T4P expression and/or assembly. First, whole cell lysates were analyzed by immunoblotting with anti-PilA antibodies^38^. The Δ*epsW* mutant was found to express PilA at a similar level as other strains (Fig. 3) known to express PilA at the wild-type level^12^,^39^. These results indicate that the Δ*epsW* mutation does not lead to defects in PilA expression. Next, the fraction that contains surface pili^39^ was subjected to immunoblotting with the same antibodies (Fig. 3). The Δ*epsW* mutant was found to be competent in the assembly of surface T4P as compared to the piliated *difE* mutant^12^. In this experiment, the *pilB* mutant was used as a negative control because it cannot assemble T4P despite its ability to express PilA^39^,^40^,^41^. The function of EpsW in *M. xanthus* EPS regulation is therefore not upstream of T4P. Instead these results are consistent with EpsW being downstream of DifE in *M. xanthus* EPS regulation as a substrate for its phosphorylation.

### EpsW is phosphorylated by the DifE kinase *in vitro*

**Figure 4:**
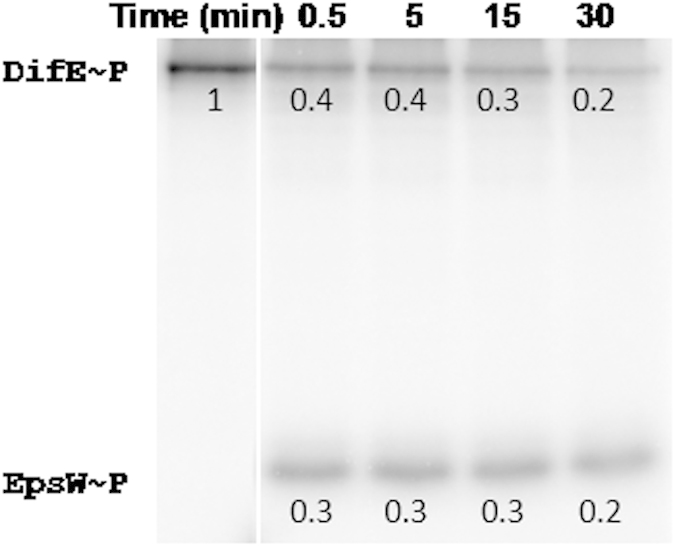
*In vitro* phosphotransfer from DifE~P to EpsW. Prephosphorylated DifE~P labeled with [γ-32 P]ATP was mixed with equimolar amounts of EpsW and incubated for the indicated times in minutes (min). Samples were separated by SDS-PAGE and analyzed by phosphorimaging as described in Methods. The number below each band is the relative radioactivity as normalized to that of DifE~P without EpsW as loaded in the first lane. The position of each protein is indicated on the left.

We determined if EpsW is a target for DifE phosphorylation using purified proteins expressed in *Escherichia coli* (Fig. 4). EpsW with a 6 × His tag at its N-terminus was purified as described in Methods. DifE was purified and phosphorylated using [γ-^32^P] ATP as previously described^11^. Phosphorylated DifE (DifE~P) was then mixed with an equimolar EpsW and incubated for 0.5, 5, 15 and 30 minutes before the reaction was terminated. These samples were separated by SDS-PAGE and phosphorylation was monitored by phosphorimaging. As shown in Fig. 4, DifE~P lost ~60% of its phosphate to EpsW in half a minute with the concurrent appearance of EpsW~P. Over the course of 30 minutes, the amount of DifE~P gradually decreased although no further increase in EpsW phosphorylation was observed. It is known that DifE~P by itself is stable as has been observed for other phosphorylated HKs^11^. These results therefore indicated that additional phosphate transfer from DifE~P to EspW occurred and the lack of further increase in the level of EpsW~P may be explained by dephosphorylation. The results here clearly demonstrate that EpsW is a substrate of DifE phosphorylation. Because DifD is also phosphorylated by DifE~P^11^, this is therefore a case where one HK phosphorylates multiple RRs^20^,^21^,^42^. These results (Figs 1, 2, 3, 4) are consistent with the notion that EspW regulates *M. xanthus* EPS production in the same signal transduction system as DifE and DifD.

### Phosphorylation of EpsW and DifE is likely critical for their function

The phosphorylation of and phosphate transfer between DifE and EpsW are presumed to be important for their role in *M. xanthus* EPS regulation. To examine this experimentally, the conserved histidine residue (H47) for autophosphorylation in DifE^39^,^43^ was mutated to an alanine. This H47A mutant allele of *difE* (*difE*^*H47A*^) was used to replace its wild-type counterpart at its chromosomal location (see Methods). As shown in Fig. 2, this *difE*^*H47A*^ mutant resembled a *difE* deletion stain as both showed a lack of S motility and EPS production. This observation shows that the autophosphorylation of DifE, thus its histidine kinase activity by extension, is likely critical for its function as a regulator of EPS production in *M. xanthus*.

Next, aspartate 58 (D58) of EpsW, the consensus phosphorylation site (Fig. 1), was mutated to an alanine (A) to examine if the phosphorylation of EpsW is required for EPS production. The plasmid with the D58A mutation of *epsW* (*epsW*^*D58A*^) was examined for complementation of Δ*epsW*. As shown in Fig. 2, unlike the wild-type, the *epsW*^*D58A*^ mutant allele failed to complement Δ*epsW* in S motility and EPS. These mutagenesis results argue that the phosphorylation events involving DifE and EpsW play key roles in EPS regulation and that the phosphorylated DifE and EpsW are the active forms of these proteins in EPS regulation in *M. xanthus*.

### EpsW may function as an intermediary in a multistep phosphorrelay

Our results thus far demonstrate that EpsW accepts phosphate from and functions downstream of DifE in the EPS regulatory pathway. Because EpsW is a CheY-like single domain RR, it may regulate the activity of an EPS biosynthetic enzyme by direct protein-protein interaction as does CheY with motor switch proteins^44^,^45^,^46^. As discussed above, the *epsW*^*D58A*^ mutation led to the same EPS^-^ phenotype as the Δ*epsW* null allele. The phosphorylation therefore would presumably convert EpsW to a signaling conformation to activate its target enzyme by physical interaction. If this is true, a mutation may convert EpsW to an active conformation without the need for phosphorylation. Alternatively, EpsW~P may pass its phosphate to another protein in a multi-step phosphorelay^47^. In this scenario, EpsW would act as an intermediary in a multistep phosphorelay to deliver the phosphate from its kinase to a phosphate acceptor downstream. As a consequence, the phosphorylation of EpsW would be required to signal downstream in this case.

**Figure 5:**
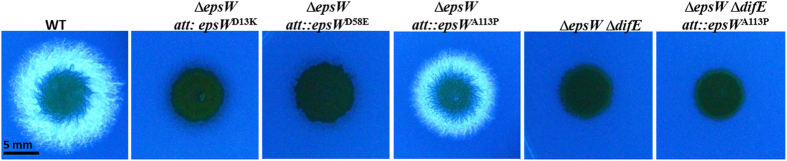
EPS production of *epsW* point mutants. EPS production was analyzed as in Fig. 2. Strains used were: DK1622 (wild type, WT), YZ1832 (Δ*epsW att*::*epsW*^D13K^), YZ1834 (Δ*epsW att*::*epsW*^D58E^), YZ1836 (Δ*epsW att*::*epsW*^Q113P^), YZ1840 (Δ*difE* Δ*epsW*), YZ1842 (Δ*difE* Δ*epsW att*::*epsW*^Q113P^).

For a RR not involved in multistep phosphorelay such as CheY, CheB or NtrC, replacing its phosphorylated aspartate residue with a glutamate (E) is known to convert it to a constitutively active form^48^,^49^,^50^,^51^. This would be the equivalent of a D58E mutation in *epsW* (Fig. 1). In addition, the equivalents of D13K and Q114P mutations of *epsW* produced constitutively active CheY proteins that favor clockwise (CW) flagellar rotation in *E. coli*^51^. In other systems, these RR mutations can also bypass the requirement for their cognate kinases^51^. These three substitutions were made in EpsW and they were examined first for complementation of the Δ*epsW* mutation (Fig. 5). Somewhat unexpectedly, both the *epsW*^*D13K*^ and *epsW*^*D58E*^ alleles failed to restore EPS production to the Δ*espW* mutant, indicating that they are not gain-of-function mutations leading to constitutively active proteins. The *epsW*^*Q114P*^ allele did restore EPS production to the Δ*epsW* strain (Fig. 5). When this allele was transformed into a Δ*difE* Δ*epsW* double mutant, however, the resulting strain mirrored the EPS^-^ phenotype of the parental strain. The phosphorylation of EpsW^Q114P^ is therefore likely required for its function in *M. xanthus* EPS regulation. These results here suggest that EpsW is likely not the terminal acceptor of phosphates from its kinase in EPS regulation. Instead, it may function as an intermediary to donate phosphates downstream in a multistep phosphorelay.

## Discussion

In this study, we searched for the previously postulated RR that functions downstream of DifE as the substrate of this kinase. Bioinformatics analysis using the specificity determinant residues in DifD led to EpsW as a possible candidate (Fig. 1). Genetics experiments demonstrated that *epsW* is indispensable for EPS production in *M. xanthus* (Fig. 2). Studies with purified proteins showed that EpsW accepted phosphate from DifE~P (Fig. 4). These results indicated that EpsW is part of the Dif pathway for the regulation of EPS in *M. xanthus*. Interestingly, observations with specific *epsW* mutations suggest that EpsW is unlikely the terminal phosphate acceptor in the Dif pathway (Fig. 5). Here we propose that EpsW is downstream of the DifE kinase as a target of its phosphorylation. Instead of the terminal RR of the pathway, EpsW is more likely an intermediary in a multistep histidine-aspartate phosphorelay that regulates EPS production in *M. xanthus*.

There are a few lines of evidence that EpsW is a target of DifE and functions downstream of this kinase. The rapid dephosphorylation of DifE~P and the simultaneous phosphorylation of EpsW (Fig. 4) clearly demonstrate that EpsW is an authentic and cognate substrate for the DifE kinase^21^,^42^. As a substrate, EpsW could function downstream of DifE or as another phosphate sink like DifD^11^,^16^. The deletion of *epsW* led to EPS^-^ (Fig. 2), the opposite of the EPS overproduction phenotype of a *difD* mutant^13^; this supports EpsW being downstream of DifE. Additional corroborations come from the observation that EpsW does not affect T4P production (Fig. 3) and that the *epsW*^*Q114P*^ mutation supported EPS production only in a *difE* wild-type background (Fig. 5). Mutagenesis of the consensus phosphorylation residues in DifE and EpsW (Fig. 2) lends support to the notion that the phosphorylation involving DifE and EpsW is key to their role in EPS regulation and that their phosphorylated forms apparently promote EPS production. We propose that signals from T4P^16^ stimulate DifE autophosphorylation which leads to the increase in the levels of phosphorylated EpsW to enhance EPS production in *M. xanthus*.

There are at least two possible scenarios how EpsW, a single-domain RR, may signal EPS downstream. Like CheY in chemotaxis^51^, EpsW could function as the terminal phosphate acceptor of the Dif regulatory pathway. That is, the activated form of EpsW, i.e., EpsW~P, would directly interact with a target protein to affect EPS production without further phosphate transfer. Alternatively, EpsW~P may passs its phosphate to proteins downstream in a phosphorelay as examplified by the sporulation regulatory phosphorelay in *B. subtilis*^47^,^52^. It is well known that a terminal RR can be converted to a constitutively active form by the substitution of either of two amino acid^51^. However, their equivalent mutations in EpsW, D13K and D58E, led to the lack of EPS instead of EPS overproduction (Fig. 5), arguing against EpsW as a terminal RR. On the other hand, the observations are easily explained if EpsW is in the middle of a phosphorelay. The *E. coli* CheY equivalent of EpsW^D13K^ is poorly phosphorylated and CheY^D57E^, the EpsW^D58E^ equivalent, lacks phosphorylation all together^48^. If the respective phosphorylation of EpsW^D13K^ and EpsW^D58E^ is poor and nonexistent, the flow of signals downstream will be attenuated/terminated in a phosphorelay, explaining the EPS^-^ phenotype of the *epsW*^*D13K*^ and *epsW*^*D58E*^ mutants (Fig. 5). The results with the *epsW*^*Q114P*^ allele is also consistent with a model of a multistep phosphorelay. It is known that CheY^A113P^ is phosphorylated by CheA to a similar level as the wild-type CheY in *E. coli*^48^. If EpsW^Q114P^ behaves in a similar manner, it will still be phosphorylated and able to transmit signals downstream in a phosphorelay in a *difE*-dependent manner. This explains the EPS^+^ phenotype of the *epsW*^*Q114A*^ single mutant and the EPS^-^ phenotype of the *epsW*^*Q114A*^ Δ*difE* double mutant (Fig. 5). It is cautioned, however, that inferences with these point mutations is by no means conclusive. The results here cannot eliminate EpsW as the terminal RR of the pathway without further experimental validation.

## Methods

### Growth Conditions

*M. xanthus* strains were grown and maintained at 32 °C on Casitone-yeast extract (CYE) agar plates or in CYE liquid medium^53^. XL1-Blue (Stratagene), the *E. coli* strain used for plasmid construction and protein expression, was grown and maintained at 37 °C on Luria-Bertani (LB) agar plates or in LB liquid medium^54^. Unless noted otherwise, agar plates contained 1.5% agar. Kanamycin and ampicillin were added to media at 100 μg/ml when appropriate.

### Construction of Plasmids and *M. xanthus* Strains

pWB435 and pMY45N, two plasmids for allelic replacement of *epsW* and *difE*, were constructed based the vector pMY7. To construct pMY7, a PCR fragment containing 26 base pairs (bp) upstream and 24 bp downstream of the coding sequence of *galK* was amplified from *Aeromonas hydrophila* str. 1719:1^55^ (Stephen A. Smith, Virginia Tech). This fragment was inserted at the tandem ApoI sites of pZErO-2 (Invitrogen) downstream of and in the same orientation as the kan^R^ gene. This vector allows gene replacement in *M. xanthus* through positive and negative selection using kanamycin and galactose, respectively^56^,^57^. An *epsW* in-frame deletion fragment was generated by a two-step overlap PCR as described previously^13^,^58^. This fragment, which deleted codons 23–121 of *epsW*, was cloned into the EcoRV site of pMY7 to create pWB435. To create a plasmid for allelic exchange of *difE* with the *difE*^*H47A*^ mutant allele, a two-step overlap PCR technique utilizing overlapping mutagenic primers^59^ was used to amplify a fragment approximately 650 bp upstream and downstream of the 47^th^ codon of *difE*. This fragment was cloned into the HindIII and XbaI sites of pMY7 to create pMY45N.

Five plasmids with the wild-type and various mutant alleles of *epsW* were constructed using pWB425 as a vector^58^,^60^. pWB425 is able to integrate at the Mx8 phage attachment site and express genes ectopically in *M. xanthus*. pWB427 contains within the EcoRV site of pWB425 a PCR fragment 50 bp upstream and 3 bp downstream of the coding region of the wild-type *epsW*. pWB427 was then used to construct pWB441, pWB442, pWB444 and pWB447 which contain the D13K, D58A, Q113P and D58E substitutions in *epsW*, respectively. These mutations were introduced by a two-step overlap PCR technique^59^.

All strains used in this study are isogenic to the wild-type strain DK1622^61^. YZ603 (Δ*difE*), DK10407 (Δ*pilA*::tet) and DK10416 (Δ*pilB*) have been described previously^13^,^38^. The Δ*epsW* deletion mutant YZ1830 was constructed from DK1622 using pWB435 and a positive-negative selection method^13^,^57^,^62^. The Δ*difE* Δ*epsW* double deletion strain YZ1640 was constructed similarly using pWB435 and YZ603 (Δ*difE*) as the parental strain. To create YZ1369 (*difE*^*H47A*^), pMY45N was used to replace the wild-type *difE* allele in DK1622. The strains YZ1831 (Δ*epsW attB*::*epsW*), YZ1832 (Δ*epsW attB*::*epsW*^*D13K*^), YZ1833 (Δ*epsW attB*::*epsW*^*D58A*^), YZ1834 (Δ*epsW attB*::*epsW*^*D58E*^), YZ1836 (Δ*epsW attB*::*epsW*^*Q113P*^) were constructed by electroporation of pWB427, pWB441, pWB442, pWB444 and pWB447 into YZ1830 (Δ*epsW*), respectively. YZ1642 (Δ*difE* Δ*epsW attB*::*epsW*^*Q113P*^) was constructed by electroporation of pWB447 into YZ1640 (Δ*difE* Δ*epsW*).

### Examination of S motility and EPS Production

Strains to be examined for S motility and EPS production were grown in CYE liquid medium, harvested during exponential growth and resuspended in MOPS (morpholinopropanesulfonic acid) buffer (10 mM MOPS [pH7.6], 2 mM MgSO_4_) at 5×10^9^ cells/ml. For analysis of S motility, 5 μl of this cell suspension was spotted onto CYE plates containing 0.4% agar and allowed to incubate at 32 °C for 5 days before documentation. For examination of EPS production, plates containing the fluorescent dye Calcofluor white were utilized^63^,^64^. Briefly, 5 μl of cells at 5 × 10^9^ cells/ml was spotted onto CYE plates supplemented with the dye Calcofluor white at 50 μg/ml which EPS^+^ strains bind. Fluorescence upon exposure to ~365 nm UV was documented after incubation at 32 °C for 5 days.

### Analysis of PilA expression and assembly of T4P

For the examination of PilA expression, whole cell lysates from 5 × 10^7^ cells were separated by 15% SDS-PAGE and analyzed by immunoblotting with anti-PilA antibodies^38^ as the primary antibody and goat-anti-rabbit antibodies conjugated to alkaline phosphatase as the secondary antibody (Santa Cruz Biotechnology). To examine strains for the assembly of T4P, pili isolated from 5 × 10^8^ cells were prepared by shearing and a differential precipitation as previously described^12^,^39^. Briefly, surface pili were sheared from cell suspensions by vortexing for 3 minutes. Intact cells were removed by two rounds of centrifugation at 16,000×g for 10 minutes. MgCl_2_ was added to the supernatant at a final concentration of 100 mM. After incubation on ice for 60 min, the pilus fraction was collected by centrifugation at 16,000 g for 15 min and resuspended in SDS-PAGE loading buffer prior to SDS-PAGE and immunoblotting analysis with anti-PilA antibodies.

### Protein expression and purification

To express and purify EpsW, plasmid pWB717 was constructed by cloning the entire coding region of *epsW* (except the start codon) into the BamHI and HindIII sites of pQE30 (Qiagen). This fused an N-terminal 6 × His tag to EpsW. For the purification of 6 × His-EpsW, a procedure similar to that of 6 × His-DifD was employed^11^. Briefly, XL1-Blue containing pWB717 was grown at 37 °C to an OD_600_ of 0.5–0.6 in 1 L of LB media with ampicillin. IPTG (isopropyl-β-D-1-thiogalactopyranoside) was added to 0.1 mM to induce at 19 °C for approximately 15 hrs. Cells were harvested by centrifugation and stored at −80 °C until needed. Cells suspended in 20 ml of binding buffer (20 mM NaH2PO4, 500 mM NaCl, 25 mM imidazole, 10% glycerol, pH 7.4) were lysed by two consecutive passes at 18,000 p.s.i. through a French press (Thermo Scientific). Cellular debris was removed by centrifugation at 16,000×g for 30 minutes, the supernatant was centrifuged at 100,000×g for 60 minutes. The supernatant was filtered (0.45 μm) and loaded on a 5 ml HisTrap FF (GE Healthcare) column using an Akta Prime chromatography system (GE Healthcare). Elution was performed using a 25–500 mM imidazole gradient of 50 ml. Fractions were analyzed by SDS-PAGE and those judged to contain EpsW at greater than 90% purity were buffer exchanged into storage buffer (10 mM Tris-Cl [pH 7.5], 25 mM KCl, 5 mM MgCl_2_, 0.5 mM EDTA, 1 mM β-mercaptoethanol [β-ME], and 10% glycerol) using a HiTrap desalting column (GE Healthcare). Protein concentration was measured using the Bio-Rad protein assay with BSA as the standard. Protein stocks were adjusted to 10 mM in storage buffer and stored at −80 °C until needed. 6 × His-DifE were purified as previously described^11^.

### Phosphotransfer assays

Phosphotransfer assays were performed similarly as previously described^11^. Briefly, DifE was prephosphorylated in 1×kinase buffer (10 mM HEPES [pH 8], 25 mM KCl, 5 mM MgCl_2_, 0.5 mM EDTA, 1 mM β-ME and 10% glycerol) with 15 μCi of [γ-^32^P]ATP (3,000 Ci/mmol, 10 mCi/ml) for 45 min at room temperature. The reaction was quenched by addition of 10,000-fold excess of unlabeled ATP to a final concentration of 0.5 mM. DifE~P was mixed with EpsW in 1×kinase buffer to give final concentrations of 1 μM for each protein and allowed to incubate for the specified times. The reactions were stopped by addition of 4×SDS-PAGE loading buffer (250 mM Tris-Cl [pH 6.8], 8% SDS, 0.004% bromphenol blue, 40% glycerol, 20% β-ME, 200 mM EDTA). Samples were then separated by 15% SDS-PAGE and the gel was dried on filter paper and exposed to a phosphor screen for approximately 15 hr. Imaging of the phosphor screen was performed using a Typhoon Trio (GE Healthcare) scanner. Image analysis and quantification was performed using ImageQuant TL (GE Healthcare).

## Additional Information

**How to cite this article**: Black, W. P. *et al*. The orphan response regulator EpsW is a substrate of the DifE kinase and it regulates exopolysaccharide in *Myxococcus xanthus*. *Sci. Rep*. **5**, 17831; doi: 10.1038/srep17831 (2015).

## References

[b1] Zusman D. R., Scott A. E., Yang Z. & Kirby J. R. Chemosensory pathways, motility and development in *Myxococcus xanthus*. Nat Rev Microbiol 5, 862–872 (2007). 17922045 10.1038/nrmicro1770

[b2] Yang Z. & Higgs P. I. Myxobacteria: genomics, cellular and molecular biology. (Caister Academic Press, 2014).

[b3] Remis J. P. . Bacterial social networks: structure and composition of *Myxococcus xanthus* outer membrane vesicle chains. Environ Microbiol 16, 598–610 (2014). 23848955 10.1111/1462-2920.12187 PMC4234120

[b4] Kaiser D., Robinson M. & Kroos L. Myxobacteria, polarity, and multicellular morphogenesis. Cold Spring Harb Perspect Biol 2, a000380 (2010). 20610548 10.1101/cshperspect.a000380 PMC2908774

[b5] Zhang Y., Ducret A., Shaevitz J. & Mignot T. From individual cell motility to collective behaviors: insights from a prokaryote, *Myxococcus xanthus*. *FEMS* Microbiol Rev 36, 149–164 (2012). 10.1111/j.1574-6976.2011.00307.x 22091711

[b6] Mauriello E. M., Mignot T., Yang Z. & Zusman D. R. Gliding motility revisited: how do the myxobacteria move without flagella ? Microbiol Mol Biol Rev 74, 229–249 (2010). 20508248 10.1128/MMBR.00043-09 PMC2884410

[b7] Spormann A. M. Gliding motility in bacteria: insights from studies of *Myxococcus xanthus*. Microbiol Mol Biol Rev 63, 621–641 (1999). 10477310 10.1128/mmbr.63.3.621-641.1999 PMC103748

[b8] Yang Z., Li C. Y., Friedrich C. & Sogaard-Andersen L. In Myxobacteria: Genomics, Cellular and Molecular Biology (eds Yang Z. & Higgs P. I.) Ch. 10, 183–198 (Caister Academic Press, 2014).

[b9] Yang Z., Geng Y., Xu D., Kaplan H. B. & Shi W. A new set of chemotaxis homologues is essential for *Myxococcus xanthus* social motility. Mol. Microbiol. 30, 1123–1130. (1998). 9988486 10.1046/j.1365-2958.1998.01160.x

[b10] Yang Z. & Li Z. Demonstration of interactions among *Myxococcus xanthus* Dif chemotaxis-like proteins by the yeast two-hybrid system. Arch. Microbiol. 183, 243–252 (2005). 15770502 10.1007/s00203-005-0767-8

[b11] Black W. P., Schubot F. D., Li Z. & Yang Z. Phosphorylation and dephosphorylation among Dif chemosensory proteins essential for exopolysaccharide regulation in *Myxococcus xanthus*. Journal of bacteriology 192, 4267–4274 (2010). 20543066 10.1128/JB.00403-10 PMC2937368

[b12] Yang Z. . *Myxococcus xanthus dif* genes are required for biogenesis of cell surface fibrils essential for social gliding motility. J. Bacteriol. 182, 5793–5798. (2000). 11004179 10.1128/jb.182.20.5793-5798.2000 PMC94702

[b13] Black W. P. & Yang Z. *Myxococcus xanthus* chemotaxis homologs DifD and DifG negatively regulate fibril polysaccharide production. J. Bacteriol. 186, 1001–1008 (2004). 14761994 10.1128/JB.186.4.1001-1008.2004 PMC344214

[b14] Park S. Y. . Structure and function of an unusual family of protein phosphatases: the bacterial chemotaxis proteins CheC and CheX. Mol Cell 16, 563–574 (2004). 15546616 10.1016/j.molcel.2004.10.018

[b15] Szurmant H., Muff T. J. & Ordal G. W. *Bacillus subtilis* CheC and FliY are members of a novel class of CheY-P-hydrolyzing proteins in the chemotactic signal transduction cascade. *The* Journal of biological chemistry 279, 21787–21792 (2004). 14749334 10.1074/jbc.M311497200

[b16] Black W. P., Xu Q. & Yang Z. Type IV pili function upstream of the Dif chemotaxis pathway in *Myxococcus xanthus* EPS regulation. Mol. Microbiol. 61, 447–456 (2006). 16856943 10.1111/j.1365-2958.2006.05230.x

[b17] Riepl H. . Interaction of CheY2 and CheY2-P with the cognate CheA kinase in the chemosensory-signalling chain of *Sinorhizobium meliloti*. Molecular microbiology 69, 1373–1384 (2008). 18573176 10.1111/j.1365-2958.2008.06342.x

[b18] Krell T. . Diversity at its best: bacterial taxis. Environ Microbiol 13, 1115–1124 (2011). 21087385 10.1111/j.1462-2920.2010.02383.x

[b19] Porter S. L., Wadhams G. H. & Armitage J. P. Signal processing in complex chemotaxis pathways. Nat Rev Microbiol 9, 153–165 (2011). 21283116 10.1038/nrmicro2505

[b20] Capra E. J. & Laub M. T. Evolution of two-component signal transduction systems. Annu Rev Microbiol 66, 325–347 (2012). 22746333 10.1146/annurev-micro-092611-150039 PMC4097194

[b21] Laub M. T. & Goulian M. Specificity in two-component signal transduction pathways. Annu. Rev. Genet. 41, 121–145 (2007). 18076326 10.1146/annurev.genet.41.042007.170548

[b22] Huynh T. N., Chen L. L. & Stewart V. Sensor-response regulator interactions in a cross-regulated signal transduction network. Microbiology (2015). 10.1099/mic.0.000092 PMC4635505 25873583

[b23] Lopez-Redondo M. L. . Environmental control of phosphorylation pathways in a branched two-component system. Molecular microbiology 78, 475–489 (2010). 20979345 10.1111/j.1365-2958.2010.07348.x

[b24] Lunt B. . Inference of direct residue contacts in two-component signaling. Methods in enzymology 471, 17–41 (2010). 20946840 10.1016/S0076-6879(10)71002-8

[b25] Procaccini A., Lunt B., Szurmant H., Hwa T. & Weigt M. Dissecting the specificity of protein-protein interaction in bacterial two-component signaling: orphans and crosstalks. PloS one 6, e19729 (2011). 21573011 10.1371/journal.pone.0019729 PMC3090404

[b26] Szurmant H. . Co-evolving motions at protein-protein interfaces of two-component signaling systems identified by covariance analysis. Biochemistry 47, 7782–7784 (2008). 18588317 10.1021/bi8009604 PMC2830073

[b27] Szurmant H. & Hoch J. A. Interaction fidelity in two-component signaling. Current opinion in microbiology 13, 190–197 (2010). 20133181 10.1016/j.mib.2010.01.007 PMC2847666

[b28] Weigt M., White R. A., Szurmant H., Hoch J. A. & Hwa T. Identification of direct residue contacts in protein-protein interaction by message passing. Proc. Natl. Acad. Sci. USA 106, 67–72 (2009). 19116270 10.1073/pnas.0805923106 PMC2629192

[b29] Capra E. J. . Systematic dissection and trajectory-scanning mutagenesis of the molecular interface that ensures specificity of two-component signaling pathways. PLoS Genet 6, e1001220 (2010). 21124821 10.1371/journal.pgen.1001220 PMC2991266

[b30] Skerker J. M. . Rewiring the specificity of two-component signal transduction systems. Cell 133, 1043–1054 (2008). 18555780 10.1016/j.cell.2008.04.040 PMC2453690

[b31] Casino P., Rubio V. & Marina A. Structural insight into partner specificity and phosphoryl transfer in two-component signal transduction. Cell 139, 325–336 (2009). 19800110 10.1016/j.cell.2009.08.032

[b32] Goldman B. S. . Evolution of sensory complexity recorded in a myxobacterial genome. Proc. Natl. Acad. Sci. USA 103, 15200–15205 (2006). 17015832 10.1073/pnas.0607335103 PMC1622800

[b33] Shi X. . A bioinformatics and experimental analysis of proteins of two-component systems in *Myxococcus xanthus*. Journal of bacteriology (2007). 10.1128/JB.01502-07 PMC2223698 17993514

[b34] Lu A. . Exopolysaccharide biosynthesis genes required for social motility in *Myxococcus xanthus*. Mol. Microbiol. 55, 206–220 (2005). 15612929 10.1111/j.1365-2958.2004.04369.x

[b35] Shi W. & Zusman D. R. The two motility systems of *Myxococcus xanthus* show different selective advantages on various surfaces. Proc. Natl. Acad. Sci. USA 90, 3378–3382 (1993). 8475084 10.1073/pnas.90.8.3378 PMC46303

[b36] Moak P. L., Black W. P., Wallace R. A., Li Z. & Yang Z. The Hsp70-like StkA functions between T4P and Dif signaling proteins as a negative regulator of exopolysaccharide in *Myxococcus xanthus*. PeerJ 3, e747 (2015). 25674362 10.7717/peerj.747 PMC4319316

[b37] Wallace R. A., Black W. P., Yang X. & Yang Z. A CRISPR with Roles in *Myxococcus xanthus* Development and Exopolysaccharide Production. Journal of bacteriology 196, 4036–4043 (2014). 25201946 10.1128/JB.02035-14 PMC4248871

[b38] Wu S. S. & Kaiser D. Regulation of expression of the *pilA* gene in *Myxococcus xanthus*. Journal of bacteriology 179, 7748–7758. (1997). 9401034 10.1128/jb.179.24.7748-7758.1997 PMC179738

[b39] Wall D., Wu S. S. & Kaiser D. Contact stimulation of Tgl and type IV pili in *Myxococcus xanthus*. J. Bacteriol. 180, 759–761. (1998). 9457887 10.1128/jb.180.3.759-761.1998 PMC106951

[b40] Jakovljevic V., Leonardy S., Hoppert M. & Sogaard-Andersen L. PilB and PilT are ATPases acting antagonistically in type IV pilus function in *Myxococcus xanthus*. Journal of bacteriology 190, 2411–2421 (2008). 18223089 10.1128/JB.01793-07 PMC2293208

[b41] Wu S. S., Wu J. & Kaiser D. The *Myxococcus xanthus pilT* locus is required for social gliding motility although pili are still produced. Mol. Microbiol. 23, 109–121. (1997). 9004225 10.1046/j.1365-2958.1997.1791550.x

[b42] Skerker J. M., Prasol M. S., Perchuk B. S., Biondi E. G. & Laub M. T. Two-component signal transduction pathways regulating growth and cell cycle progression in a bacterium: a system-level analysis. PLoS Biol. 3, e334 (2005). 16176121 10.1371/journal.pbio.0030334 PMC1233412

[b43] Hess J. F., Bourret R. B. & Simon M. I. Histidine phosphorylation and phosphoryl group transfer in bacterial chemotaxis. Nature 336, 139–143 (1988). 3185734 10.1038/336139a0

[b44] Stock D., Namba K. & Lee L. K. Nanorotors and self-assembling macromolecular machines: the torque ring of the bacterial flagellar motor. Curr Opin Biotechnol 23, 545–554 (2012). 22321941 10.1016/j.copbio.2012.01.008

[b45] Sarkar M. K., Paul K. & Blair D. Chemotaxis signaling protein CheY binds to the rotor protein FliN to control the direction of flagellar rotation in *Escherichia coli*. Proc. Natl. Acad. Sci. USA 107, 9370–9375 (2010). 20439729 10.1073/pnas.1000935107 PMC2889077

[b46] Paul K., Brunstetter D., Titen S. & Blair D. F. A molecular mechanism of direction switching in the flagellar motor of *Escherichia coli*. Proc. Natl. Acad. Sci. USA 108, 17171–17176 (2011). 21969567 10.1073/pnas.1110111108 PMC3193218

[b47] Hoch J. A. Two-component and phosphorelay signal transduction. Current opinion in microbiology 3, 165–170 (2000). 10745001 10.1016/s1369-5274(00)00070-9

[b48] Bourret R. B., Drake S. K., Chervitz S. A., Simon M. I. & Falke J. J. Activation of the phosphosignaling protein CheY. II. Analysis of activated mutants by 19 F NMR and protein engineering. The Journal of biological chemistry 268, 13089–13096 (1993). 8514750 PMC2892986

[b49] Bourret R. B., Hess J. F. & Simon M. I. Conserved aspartate residues and phosphorylation in signal transduction by the chemotaxis protein CheY. Proc. Natl. Acad. Sci. USA 87, 41–45 (1990). 2404281 10.1073/pnas.87.1.41 PMC53195

[b50] Klose K. E., Weiss D. S. & Kustu S. Glutamate at the site of phosphorylation of nitrogen-regulatory protein NTRC mimics aspartyl-phosphate and activates the protein. Journal of molecular biology 232, 67–78 (1993). 8331671 10.1006/jmbi.1993.1370

[b51] Smith J. G. . A search for amino acid substitutions that universally activate response regulators. Molecular microbiology 51, 887–901 (2004). 14731287 10.1046/j.1365-2958.2003.03882.x

[b52] Appleby J. L., Parkinson J. S. & Bourret R. B. Signal transduction via the multi-step phosphorelay: not necessarily a road less traveled. Cell 86, 845–848 (1996). 8808618 10.1016/s0092-8674(00)80158-0

[b53] Campos J. M. & Zusman D. R. Regulation of development in Myxococcus xanthus: effect of 3′:5′-cyclic AMP, ADP, and nutrition. Proc. Natl. Acad. Sci. USA 72, 518–522. (1975). 10.1073/pnas.72.2.518 PMC432343 164657

[b54] Miller J. H. Experiments in molecular genetics. (Cold Spring Harbor Laboratory, 1972).

[b55] Seshadri R. . Genome sequence of *Aeromonas hydrophila* ATCC 7966 T: jack of all trades. Journal of bacteriology 188, 8272–8282 (2006). 16980456 10.1128/JB.00621-06 PMC1698176

[b56] Julien B., Kaiser A. D. & Garza A. Spatial control of cell differentiation in *Myxococcus xanthus*. Proc. Natl. Acad. Sci. USA 97, 9098–9103 (2000). 10922065 10.1073/pnas.97.16.9098 PMC16828

[b57] Ueki T., Inouye S. & Inouye M. Positive-negative KG cassettes for construction of multi-gene deletions using a single drug marker. Gene 183, 153–157. (1996). 8996101 10.1016/s0378-1119(96)00546-x

[b58] Black W. P. . Isolation and characterization of a suppressor mutation that restores *Myxococcus xanthus* exopolysaccharide production. Microbiology 155, 3599–3610 (2009). 19684067 10.1099/mic.0.031070-0 PMC2879065

[b59] Black W. P., Julien B., Rodriguez E. & Yang Z. In Manual of Industrial Microbiology and Biotechnology (eds Baltz R. H., Demian A. L., & Davies J. E.) Ch. 18, 262–272 (ASM Press, 2010).

[b60] Magrini V., Creighton C. & Youderian P. Site-specific recombination of temperate *Myxococcus xanthus* phage Mx8: genetic elements required for integration. J. Bacteriol. 181, 4050–4061. (1999). 10383974 10.1128/jb.181.13.4050-4061.1999 PMC93896

[b61] Kaiser D. Social gliding is correlated with the presence of pili in *Myxococcus xanthus*. Proc. Natl. Acad. Sci. USA 76, 5952–5956. (1979). 42906 10.1073/pnas.76.11.5952 PMC411771

[b62] Kashefi K. & Hartzell P. L. Genetic suppression and phenotypic masking of a *Myxococcus xanthus frzF*^-^ defect. Mol. Microbiol. 15, 483–494. (1995). 7783619 10.1111/j.1365-2958.1995.tb02262.x

[b63] Dana J. R. & Shimkets L. J. Regulation of cohesion-dependent cell interactions in *Myxococcus xanthus*. J. Bacteriol. 175, 3636–3647 (1993). 8501067 10.1128/jb.175.11.3636-3647.1993 PMC204765

[b64] Ramaswamy S., Dworkin M. & Downard J. Identification and characterization of *Myxococcus xanthus* mutants deficient in calcofluor white binding. J. Bacteriol. 179, 2863–2871. (1997). 9139901 10.1128/jb.179.9.2863-2871.1997 PMC179047

